# Light-curing process for clear aligners’ attachment reproduction: comparison between two nanocomposites cured by the auxiliary of a new tool

**DOI:** 10.1186/s12903-022-02407-8

**Published:** 2022-09-05

**Authors:** Francesca Gazzani, Denise Bellisario, Fabrizio Quadrini, Carlotta Danesi, Andrea Alberti, Paola Cozza, Chiara Pavoni

**Affiliations:** 1 Department of Systems Medicine, University of Rome ‘Tor Vergata’, Viale Oxford 81, 00133 Rome, Italy; 2 Department of Industrial Engineering, University of Rome ‘Tor Vergata’, Rome, Italy; 3 UniCamillus International Medical University, Rome, Italy; 4 Department of Dentistry, UNSBC, Tirana, Albania; 5 Department of Faculty of Medicine and Surgery, UniCamillus International Medical University, Rome, Italy

**Keywords:** Attachments, Clear Aligners Treatment, Nanocomposite resins, LED curing-light, Surface roughness and waviness

## Abstract

**Background:**

Attachments’ configuration play an important role during Clear Aligner Treatment (CAT) for aligner retention and control of movements planned. The aims were to compare the macroscopic morphology of attachments reproduced with flowable (FNC) and conventional (CNC) composites and the effects on them of two light-guide tips with different dimensions.

**Methods:**

4 resin casts derived from the initial scan of the same patient were obtained. 10 vestibular attachments were replaced on both upper and lower arches of each model with CNC (Models A, B) and FNC (Models C, D). Each composite was cured by means of the same LED lamp with both regular light-guide (Models A, B) and push and light tool® (Models C, D). The 80 attachments were qualitative analyzed by means of a digital stereo microscope. Surface roughness and waviness measurements were assessed by contact probe surface profiler (TalySurf CLI 2000; Taylor Hobson, Leicester, United Kingdom). Statistical analysis was performed with independent samples t-tests. Significance was established at the *P* < 0.05 level.

**Results:**

Model A showed lower values of surface roughness (Ra − 1.41 µm, Rt − 3.46 µm) and waviness (Wa − 2.36 µm, Wt − 10.95 µm) when compared with Model C. Significant reduction of waviness (Wa − 3.85 µm, Wt − 4.90 µm) was observed on Model B when compared with Model D. Significant increase of roughness and waviness parameters (Ra 3.88 µm, Rt 21.07, Wa 2.89 µm, Wt 14.74 µm) was found when CNC sample (Model A) was cured with regular light-guide tip. Higher values (Ra 2.33 µm, Rt 24.07 µm, Wa 1.67 µm, Wt 20.79 µm) were observed after regular light-guide tips curing on FNC sample (Model C).

**Conclusions:**

CNC resins determine more regular surfaces of attachments profiles. The additional use of a smaller light- guide of the LED push and light tool® allows to improve the macroscopic morphology of the attachments and to maximize light irradiance delivering by enhancing the polymerization process and the integrity of the features during the treatment.

## Background

Clear Aligners Treatment (CAT) require the application of specific auxiliaries, known as attachments, on tooth surfaces to enhance aligner retention and more predictable tooth movements [1–3]. The presence of these resins’ buttons maximizes the contact between aligners and tooth surfaces with an implementation of their interaction. For these reasons, their configurations play an important role during the entire orthodontic treatment. Attachments’ shape and dimension need to be maintained in order to ensure a better control of the movements planned [4–7]. During clinical practice, several factors can determine an early failure of the attachments in terms of debonding, shape modifications and loss of aligners’ fitting. Undoubtedly, the choice of the best material with ideal properties represents a relevant aspect for the maintenance and the wear performance of these auxiliary components [5]. In literature, many studies [1, 3, 5, 8] investigated the efficiency of different resin-based composites used for this clinical application. D’Antò et al. [3] concluded that composite viscosity does not have any influences on the attachments’ shape and volume. More recently, Gazzani et al. [4] comparing the mechanical properties of two nanocomposite resins with different viscosities and filler volume, pointed out that the higher viscosity of conventional composites determines greater wear performance, more suitable for clinical needs. Another relevant factor is represented by the light-curing process that is strongly related to the success of resin composite during their clinical applications [9]. Since the introduction of photopolymerizable dental composites, various technologies have been proposed for light-curing units such as UV (ultraviolet)-lights, quartz- tungsten halogen (QTH) lights, and light-emitting diodes (LEDs) [10]. Among the different light-curing systems available, new generation LEDs with high power irradiance (500–1400 mW/cm^2^) seem to be the best technology [11–14]. As a matter of fact, a sufficient delivering irradiance output during curing process is mandatory to ensure the longevity of composite. As shown in some studies [15, 16] the diameters of light-guide tip can affect the polymerization of light cured composite. Oesterle et al. [16] concluded that bond strengths tend to decrease with larger light-guide tip with negative effects on bracket bonding, whereas smaller light-guide tips have been found to increase the light concentration. Despite the increasing diffusion of CAT, no studies are present in literature on curing-light efficiency during attachment reproduction. The primary aim of the present study was to compare the macroscopic morphology of aligner attachments reproduced by means of two different composites with different viscosity and filler volume. Both nanocomposites were cured by means of two light-guide tips. In particular, a standard diameter of 10 mm was compared with a new LED push and light tool® characterized by a reduced tip. Thus, the second aim of the investigation was to evaluate the effects in terms of curing-light efficiency of two light-guide tips characterized by different dimensions.

## Methods

**Fig. 1:**
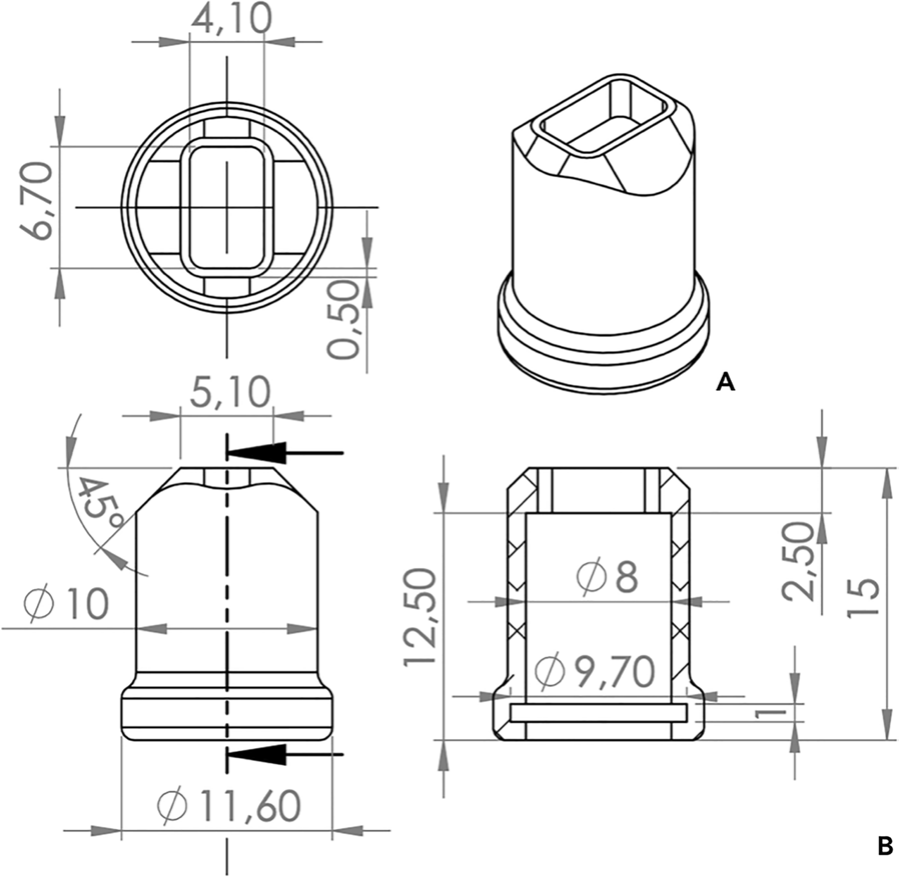
Configuration and structural details of LED push and light tool®. **A** LED push and light tool® unit **B** Dimension of different sectional areas

The sample size was estimated based on preliminary data [4]. A minimum sample of 80 attachments was needed in order to achieve 80% power, with an alpha of 5% to detect a 2 (µm) difference in Ra variable (SD 0.20 µm), and a level of significance of 0.05. An adult patient treated with Invisalign at the Department of Orthodontics of University of Rome “Tor Vergata” and presenting a permanent dentition was selected for the study. The.STL files were exported from the initial intraoral digital scans of dental arches obtained using iTero® ElementTM scanner (Align Technology, Santa Clara, CA, USA). Four resin casts derived from the initial scan (methacrylic acid esters, proprietary pigment; Form2 3D printer [Form-labs Inc., Somerville, MA, USA]) were obtained with the support of a dental laboratory. Four copies of the same initial attachments template were required from Invisalign Doctor Site to reproduce the same attachments on each resin model printed. Firstly, Assure® Plus All Surface Bonding Resin (Reliance Orthodontic Products Inc., Itasca, IL, USA) was applied and then cured by means of a LED curing light (TPC led curing light 50 N, United States) with high irradiance (1200mW/cm^2^) reading at 0 mm distance and regular light-guide tips (diameter of 10 mm). The irradiance of the lamp was checked with the integrated UV light high-power meter of the LED lamp. The LED lamp was positioned at 5 mm of distance and the curing time at 25 s as suggested by the manufacturer, with a radiation exposure of 6 J/cm^2^. A flowable (FNC, Tetric EvoFlow, Ivoclar Vivadent, Schaan, Liechtenstein) and a conventional (CNC, Transbond XT Light Cure Paste, no. 712-036, 3 M Unitek) nanocomposite with different viscosity and filler volume were selected and used for the attachments’ placement. CNC is composed of small particle sizes with a high filler volume and high viscosity of the mixture whereas FNC consists of the same small particle size with a reduced filler volume, increased resin content, and lower viscosity of the mixture [17, 18]. In terms of composition, CNC and FNC presented an inorganic percentage residue of 23% and 41%, respectively [4]. All the attachments templates were filled with the two composites by the same operator (FG). The secondary outcome was to compare the light delivering irradiance between a regular light-guide tips with a standard diameter of 10 mm and a new LED push and light tool® with reduced dimensions and rectangular section (length 4.10 mm, high 6.70 mm). In particular, the LED push and light tool® is an additional instrument mounted of the standard light-guide with a reduced rectangular section for light delivering (Fig. 1). Each template was inserted on the resin casts and 10 vestibular attachments from second molar to the canine were replaced on both upper and lower arches with CNC (Models A, B) and FNC (Models C, D). Each composite was cured by means of the same LED lamp with both the light-guides tested. The description of the model is summarized is Table 1. The curing phase was performed with an irradiance of 1200 mW/cm^2^ and the curing time step was set at 25 s with a distance of 5 mm of the LED lamp. Only when LED push and light tool®, a pressure of 1 N was applied by clinician on the attachment template using a calibrated balance. Summarizing, one of composite was tested for each model and cured with both the light-guide tips by the same operator reproducing the clinical operative procedures. At the end of the curing process, the 80 attachments were qualitative analyzed by means of a digital stereo microscope. Then, samples’ roughness and waviness were assessed by contact probe surface profiler (TalySurf CLI 2000; Taylor Hobson, Leicester, United Kingdom) using a 5 μm lateral resolution. A series of four profile for each sample was recorded and the following surface roughness and waviness measurements were evaluated with a Gaussian cutoff filter of 0.8 mm: arithmetic mean roughness value (Ra, µm), mean peak width (RSm, µm), total height of the roughness profile (Rt, µm), arithmetic mean waviness value (Wa, µm), total height of the waviness profile (Wt, µm). Appropriate statistical analysis of the results was performed with independent samples t-tests. Analysis was performed comparing surface roughness and waviness between CNC and FNC samples undergone the same curing process (Model A versus Model C; Model B versus Model C). Then the same comparison was performed on the same nanocomposite samples cured with different light-curing process (Model A versus Model B; Model C versus Model D). Significance was established at the *P* < 0.05 level.

**Table 1:** Description of resin models

Model	Number of attachments	Composite	Led Lamp
A	20	CNC	Regular light-guide
B	20	CNC	Push and light tool®
C	20	FNC	Regular light-guide
D	20	FNC	Push and light tool®

*CNC* conventional nanocomposite, *FNC* flowable nanocomposite

## Results

**Fig. 2:**
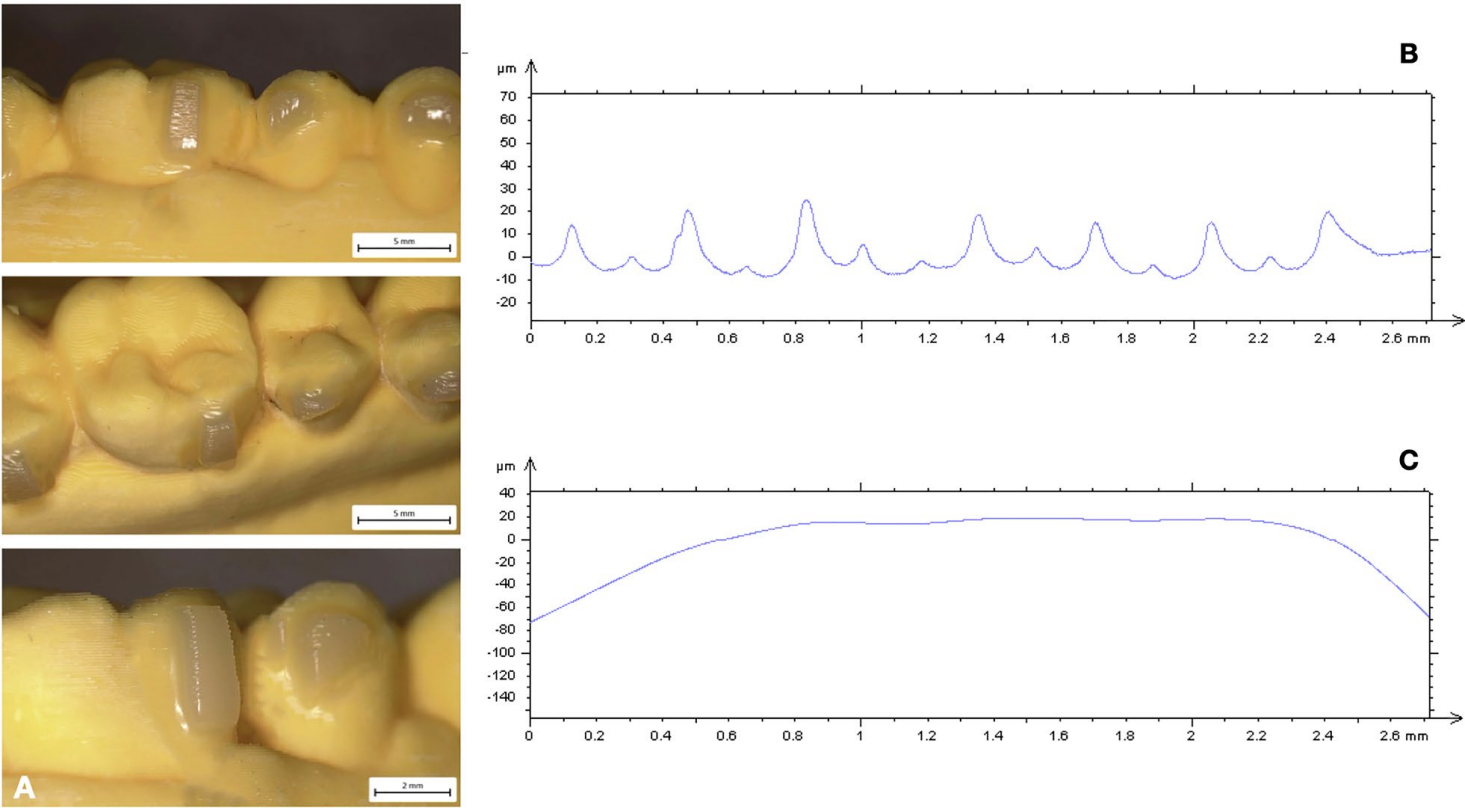
Model A (CNC + Regular light-guide). **A** Attachments surface profile **B** Surface roughness **C** Surface waviness

**Fig. 3:**
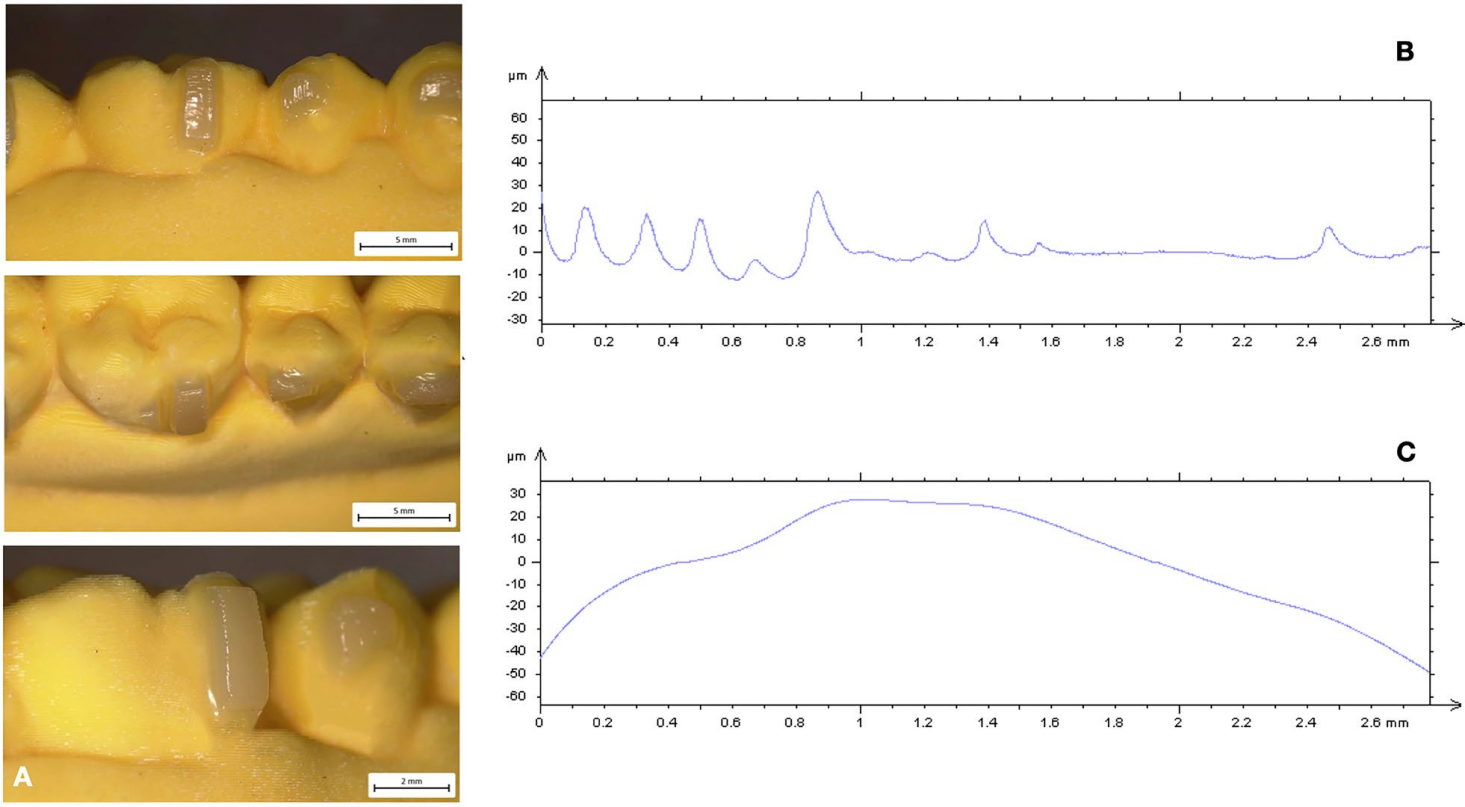
Model B (CNC + Push and light tool®). **A** Attachments surface profile **B** Surface roughness **C** Surface waviness

**Fig. 4:**
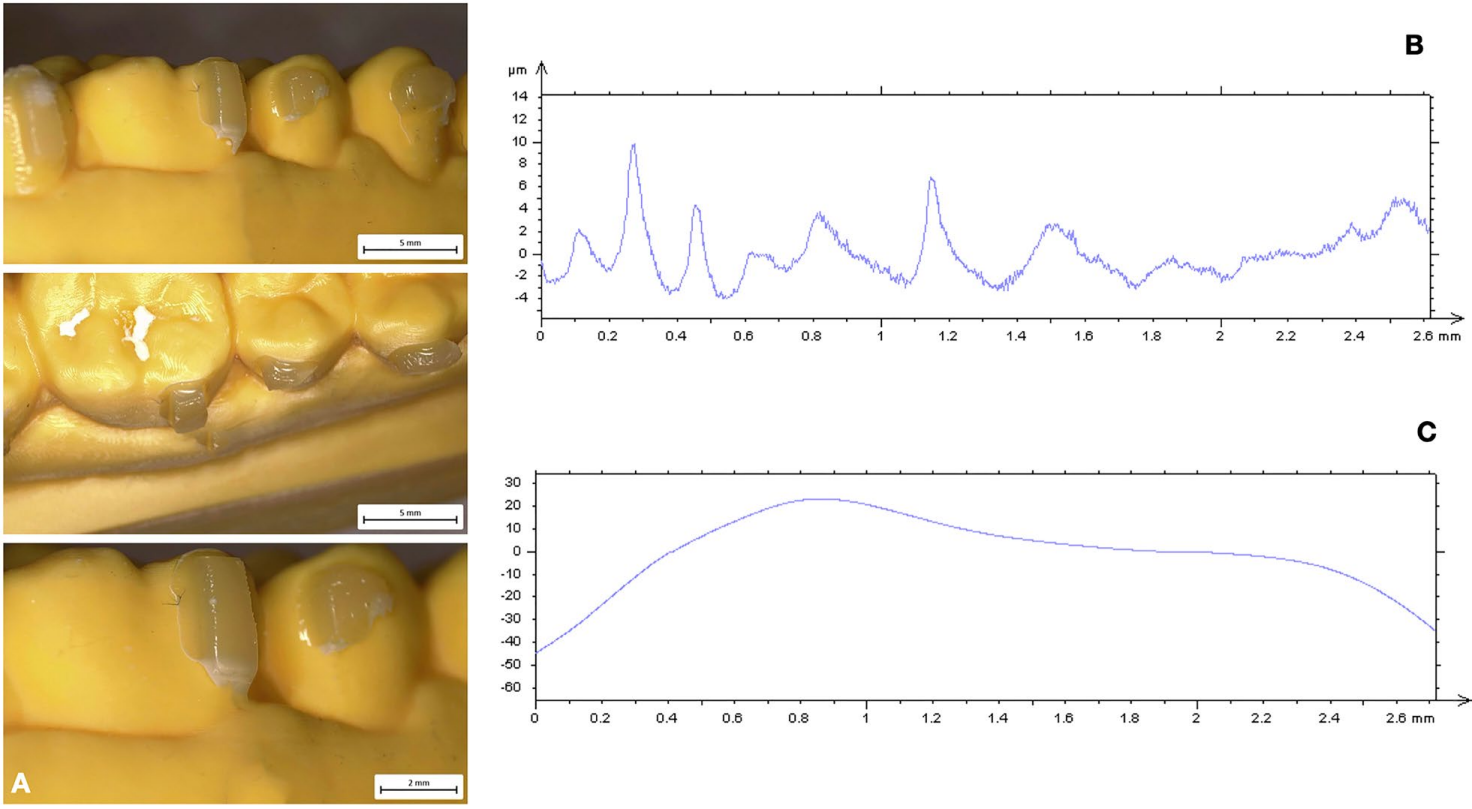
Model C (FNC + Regular light-guide). **A** Attachments surface profile **B** Surface roughness **C** Surface waviness

**Fig. 5:**
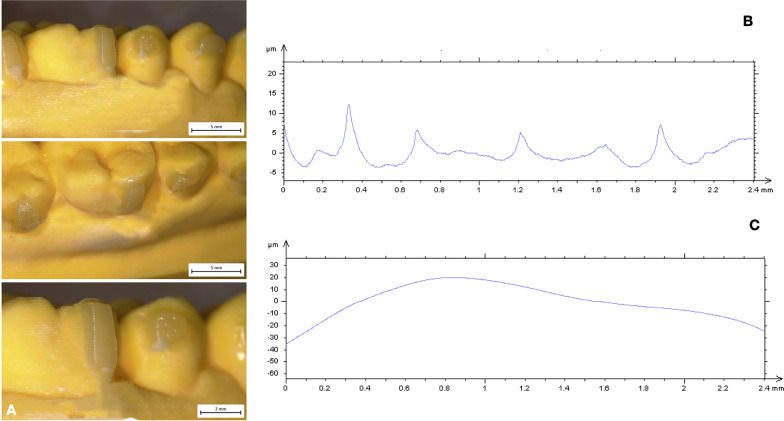
Model D (FNC + Push and light tool®). **A** Attachments surface profile **B** Surface roughness **C** Surface waviness

Description of mean values and standard deviation of surface roughness and waviness obtained by contact probe surface profiler (TalySurf CLI 2000; Taylor Hobson, Leicester, United Kingdom) are summarized in Tables 2 and 3. Results of statistical comparisons between CNC and FNC samples are shown in Tables 2. Significant differences were found between CNC and FNC samples in terms of surface roughness and waviness (Figs. 2, 3, 4, 5). Model A (Fig. 2) showed lower values of Ra (− 1.41 µm) and Rt (− 3.46 µm) when compared with Model C (Fig. 4). As for profile waviness, the same trend was observed. Statistical comparison revealed a decrease of both Wa and Wt parameters in Model A when compared with Model B (Fig. 3) (Wa − 2.36 µm, Wt − 10.95 µm). Similar results were found in the comparison between the two nanocomposites cured with the LED push and light tool® in terms of surface waviness. No significant differences were found for roughness parameters, whereas a significant reduced waviness (Wa − 3.85 µm, Wt − 4.90 µm) was observed in CNC sample (Model B, Fig. 3) when compared with FNC one (Model D, Fig. 5). Table 3 shows the results obtained from the comparison between the light guide tips for each nanocomposite resin. Findings revealed significant better values for samples cured by means of LED push and light tool®, indicating a better performance of the curing process. Both Models B and D showed significant lower values of roughness and waviness when compared respectively with Models A and C. A significant increase of Ra (3.88 µm) and Rt (21.07 µm) parameters was found in the comparison between CNC samples cured with both regular light-guide tips (Model A) and LED push and light tool® (Model B), as well as waviness measurements (Wa 2.89 µm, Wt 14.74 µm). Similarly, in FNC samples higher values of surface roughness (Ra 2.33 µm, Rt 24.07 µm) and waviness (Wa 1.67 µm, Wt 20.79 µm) were observed after regular light-guide tips curing (Model C) than LED push and light tool® sample (Model D).

**Table 2:** Descriptive statistics and statistical comparisons (independent-samples t tests) of the surface roughness and waviness measurements between CNC and FNC samples undergone the same curing process

Variables	Model A *(CNC* + *Regular light-guide)*		Model C *(FNC* + *Regular light-guide)*		Model A vs Model C		95% CI of the difference		Model B *(CNC* + *push and light tool®)*		Model D *(FNC* + *push and light tool®)*		Model B vs Model D		95% CI of the difference	
	Mean	SD	Mean	SD	Diff	Pvalue	Lower	Upper	Mean	SD	Mean	SD	Diff	Pvalue	Lower	Upper
Ra (µm)	3.77	0.95	5.20	0.63	− 1.41	0.000	0.894	1.933	1.32	0.18	1.44	0.19	− 0.13	0.028	0.0145	0.2435
RSm (µm)	0.23	0.06	0.29	0.19	− 0.06	0.185	− 0.156	0.030	0.16	0.16	0.30	0.11	− 0.13	0.004	0.046	0.231
Rt (µm)	30.83	1.48	34.29	1.61	− 3.46	0.000	− 4.461	− 2.247	9.75	1.37	10.22	0.38	− 0.47	0.473	− 0.172	1.118
Wa (µm)	6.81	0.61	9.45	1.39	− 2.63	0.000	− 3.316	− 1.958	3.92	1.14	7.78	1.11	− 3.85	0.000	3.132	4.577
Wt (µm)	37.87	2.16	48.82	1.29	− 10.95	0.000	− 12.092	− 9.811	23.13	1.58	28.03	0.75	− 4.90	0.000	4.104	5.698

*CNC* conventional nanocomposite, *FNC* flowable nanocomposite, *Ra* arithmetic mean roughness value, *RSm* mean peak width, *Rt* total height of the roughness profile, *Wa* arithmetic mean waviness value, *Wt* total height of the waviness profile, *µm* micrometer, *SD* Standard Deviations, *Diff*. Differences, *CI* Confidence interval

**Table 3:** Descriptive statistics and statistical comparisons (independent-samples t tests) of the surface roughness and waviness measurements after use of different light-guide tips

Variables	Model A *(CNC* + *Regular light-guide)*		Model B *(CNC* + *push and light tool®)*		Model A vs Model B		95% CI of the difference		Model C *(FNC* + *Regular light-guide)*		Model D *(FNC* + *push and light tool®)*		Model C vs Model D		95% CI of the difference	
	Mean	SD	Mean	SD	Diff	Pvalue	Lower	Upper	Mean	SD	Mean	SD	Diff	Pvalue	Lower	Upper
Ra (µm)	3.77	0.95	1.32	0.18	2.45	0.000	− 2.57	− 1.19	5.20	0.63	1.44	0.19	3.76	0.000	− 3.77	− 0.89
RSm (µm)	0.23	0.06	0.16	0.15	0.06	0.122	− 0.017	0.114	0.29	0.19	0.30	0.11	0.012	0.815	− 0.091	0.115
Rt (µm)	30.83	1.48	9.75	1.37	21.07	0.000	20.16	21.99	34.29	1.61	10.22	0.38	24.07	0.000	− 24.826	− 23.320
Wa (µm)	6.81	0.61	3.92	1.14	2.89	0.000	2.30	3.47	9.45	1.36	7.78	1.11	1.67	0.000	− 2.4738	− 0.8742
Wt (µm)	37.87	1.58	37.87	2.16	14.74	0.000	13.52	15.95	48.82	1.29	28.03	0.75	20.79	0.000	− 21.468	− 20.114

*CNC* conventional nanocomposite, *FNC* flowable nanocomposite, *Ra* arithmetic mean roughness value, *RSm* mean peak width, *Rt* total height of the roughness profile, *Wa* arithmetic mean waviness value, *Wt* total height of the waviness profile, *µm* micrometer, *SD* Standard Deviations, *Diff*. Differences, *CI* Confidence interval

## Discussion

**Fig. 6:**
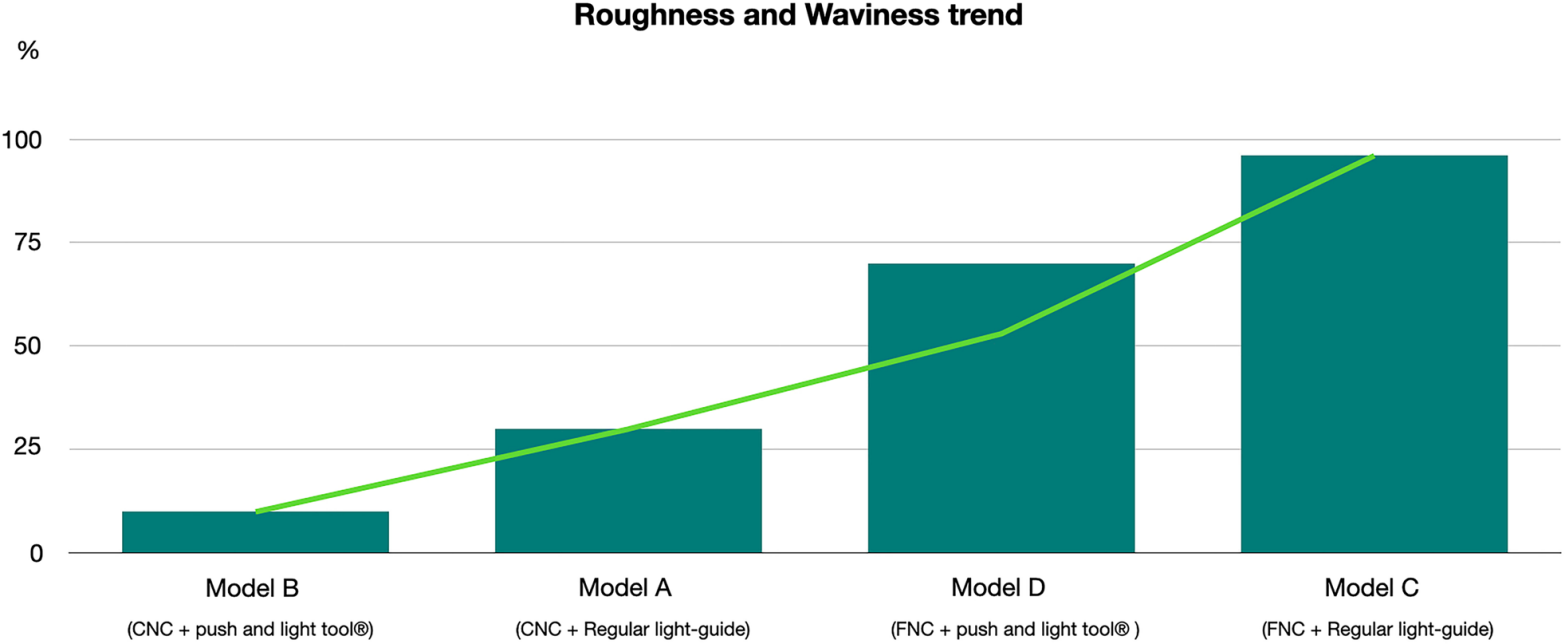
Roughness and waviness trend for all model selected

Since composite attachments represent a primary component in CAT, they should be carefully reproduced to ensure their integrity during the entire treatment [1–3, 19]. For this reason, attachment bonding procedures play a crucial role for the stability of structural shape and integrity of these features [4]. The aims of the present investigation were to compare the macroscopic morphology of attachments reproduced with two nanocomposites and the curing-light efficiency of two light-guide tips of different dimensions. Surface roughness and waviness have significant effects on structural integrity, wear behavior and surface stresses since they influence the surface properties and the interaction between attachments and clear aligners. High macroscopic accuracy plays an important role in terms of longevity of these features reducing breakage and detachment phenomena. According to a previous investigation [4], CNC resins resulted more suitable for clinical needs. The comparison of surface roughness and waviness between CNC and FNC samples undergone the same curing process (Model A versus Model C; Model B versus Model C), revealed more smoother and regular surfaces when attachments were reproduced with CNC (Model A, Fig. 2). Surface roughness values showed decreased trend in CNC samples indicating more homogeneous surfaces without scratches and macroscopic irregularities (Table 2) in agreement with the qualitative evaluation performed with the digital stereo microscope (Fig. 2). The same trend has been observed for the profile waviness with a significant decrease of Wa and Wt parameters in both CNC samples (Model A and Model B). These findings revealed a more stable and regular macroscopic morphology determining CNC characteristics more suitable to ensure a high fitting of the aligner and more performant tooth movements. Moreover, CNC presents better mechanical and wear behavior in terms of stain resistance, hardness and wear when compared with FNC [4] appearing as the best choice to grantee the maintenance of initial configuration of the attachments during the entire treatment. Lin et al. [20] comparing 1-year Invisalign aligner attachments survival concluded that attachments damage was not affected by the composite used. Their results, even if not statistically significant, confirmed a higher damage rate for flowable composite than package one after 1 year of treatment. Despite the mechanical features of CNC, also the bonding preparation process could affect the macroscopic morphology of the features. As composite dosage for attachments, FNC is easier for the presence of the injector-like design and the lower viscosity of the mixture. On the other hand, higher viscosity and density of CNC require more control and modeling ability when it is placed into the template shape (Fig. 4). The second comparison was performed on the same nanocomposite samples cured with different light-curing process (Model A versus Model B; Model C versus Model D). Findings obtained revealed a better performance of the curing process when samples were cured by means of LED push and light tool®. As shown in Table 3 both Models B (Fig. 3) and D (Fig. 5) showed significant lower values of roughness and waviness when compared respectively with Models A (Fig. 2) and C (Fig. 4), indicating more regular macroscopic surfaces of the sample (Fig. 6). The use of a smaller diameter of LED push and light tool® was useful to maximize the light delivering irradiance. Similarly, Oesterle et al. [16] testing the effect bond strength of different light-guide sizes concluded that bond strengths tend to decrease with larger diameter. Moreover, the rectangular geometry of light-guide tip (Fig. 1) allows the clinician to apply a pressure on the attachment template enhancing bond strength and macroscopic surface accuracy. As in all polymerization phases, the curing process can strongly affect attachments morphology and resistance. A biological advantage of the configuration of the LED push and light tool® consists in being a protection for the eyes against the light-induced damage. Smaller dimensions of light-guide help to reduce the dispersion and the amount of the light passing throughout operator’s eyes and the risk of tissue damage [21, 22].

## Conclusions

CNC resins determine more regular surfaces of attachments profiles granting a greater aligner fitting and an easier movements achievement. During curing process, the additional use of a smaller light-guide of the LED push and light tool® allows to improve the macroscopic morphology of the attachments and to maximize light irradiance delivering enhancing the polymerization process.

## Data Availability

All data generated or analyzed during this study are included in this published article.

## Abbreviations

CAT: Clear aligner treatment
FNC: Flowable nanocomposite
CNC: Conventional nanocomposite
LED: Light emitting diode
Ra: Arithmetic mean roughness value
RSm: Mean peak width
Rt: Total height of the roughness profile
Wa: Arithmetic mean waviness value
Wt: Total height of the waviness profile
